# MRI Relaxivity Changes of the Magnetic Nanoparticles Induced by Different Amino Acid Coatings

**DOI:** 10.3390/nano10020394

**Published:** 2020-02-24

**Authors:** Iryna Antal, Oliver Strbak, Iryna Khmara, Martina Koneracka, Martina Kubovcikova, Vlasta Zavisova, Martina Kmetova, Eva Baranovicova, Dusan Dobrota

**Affiliations:** 1 Institute of Experimental Physics, Slovak Academy of Sciences, Watsonova 47, 040 01 Kosice, Slovakia; iryna.antal@saske.sk (I.A.); irynakhmara@gmail.com (I.K.); konerack@saske.sk (M.K.); kubovcikova@saske.sk (M.K.); zavisova@saske.sk (V.Z.); 2 Biomedical Center Martin, Jessenius Faculty of Medicine in Martin, Comenius University in Bratislava, Mala Hora 4, 036 01 Martin, Slovakia; eva.baranovicova@uniba.sk; 3 Department of Medical Biochemistry, Jessenius Faculty of Medicine in Martin, Comenius University in Bratislava, Mala Hora 4, 036 01 Martin, Slovakia; martinamihalikova09@gmail.com (M.K.); dusan.dobrota@uniba.sk (D.D.)

**Keywords:** magnetic nanoparticles, maghemite, functionalisation, MRI, relaxivity, amino acid, glycine, lysine, tryptophan

## Abstract

In this study, we analysed the physico-chemical properties of positively charged magnetic fluids consisting of magnetic nanoparticles (MNPs) functionalised by different amino acids (AAs): glycine (Gly), lysine (Lys) and tryptophan (Trp), and the influence of AA–MNP complexes on the MRI relaxivity. We found that the AA coating affects the size of dispersed particles and isoelectric point, as well as the zeta potential of AA–MNPs differently, depending on the AA selected. Moreover, we showed that a change in hydrodynamic diameter results in a change to the relaxivity of AA–MNP complexes. On the one hand, we observed a decrease in the relaxivity values, *r*_1_ and *r*_2_, with an increase in hydrodynamic diameter (the relaxivity of *r*_1_ and *r*_2_ were comparable with commercially available contrast agents); on the other hand, we observed an increase in *r*_2_* value with an increase in hydrodynamic size. These findings provide an interesting preliminary look at the impact of AA coating on the relaxivity properties of AA–MNP complexes, with a specific application in molecular contrast imaging originating from magnetic nanoparticles and magnetic resonance techniques.

## 1. Introduction

The biocompatibility of nanoparticles (NPs), which is provided by a suitable coating and low toxicity of iron oxide nanoparticles (IONPs), makes them ideal candidates for applications in the biomedical fields such as magnetic resonance imaging (MRI), hyperthermia, and targeted drug delivery [1,2,3]. Currently, the combined effect of the NPs’ action, which includes targeting, MRI contrast, and thermal properties, is in high demand [4]. Magnetic nanoparticles (MNPs), in combination with proteins, are attracting significant interest in MRI-related biomedical applications [5]. Changes in the concentration levels of proteins are associated with various pathological processes, including cancer [6] and neuroinflammation [7]. Magnetic moments of MNPs shorten the relaxation time of the surrounding water protons, increasing such tissue relaxivity, and causing so-called hypointensive artefacts [4]. In addition, the protein–NP complex affects the coupling mechanism of protons’ and NPs’ magnetic moments, changing the final MRI contrast [8]. Such a change, if detectable, may provide information on the pathology in a non-invasive manner, through simple targeting and binding to the protein biomarker characteristic for the disease. 

On the other hand, the imaging of individual amino acids (AAs), as opposed to proteins, is gaining popularity because it can reveal the metabolic pathways of pathological processes. Specifically, imaging by positron emission tomography (PET) in combination with MRI techniques is becoming an increasingly in-demand application in clinical oncological research [9,10]. However, in this approach, an MRI can only provide anatomical and spatial information about the pathology (if visible), rather than information useful for the diagnostics of the pathology itself. Moreover, a conventional MRI is limited in the differentiation of tumour tissue from post-therapeutic effects following different types of therapy (i.e., immunotherapy, chemotherapy, radiosurgery, chemoradiation, or neurosurgical resection) [10]. Therefore, from the MRI perspective, the best option for MRI contrast agents can be various AA–MNP complexes, which will help to determine the changes of relaxation caused by the AA after binding to stabilised IONPs. Researchers have recently shown a rapidly increasing interest in AA–MNP complexes.

Barick et al. demonstrated synthesised superparamagnetic carboxyl decorated dendron-like Fe_3_O_4_ NPs (size approximately 10 nm) by conjugating glycine (Gly) at the interface using a facile soft-chemical approach based on Michael addition/amidation reaction [11]. These biocompatible Gly–MNPs do not have a toxic effect on L929 (primary mouse fibroblasts) or HeLa (human cervical cancer cells) and can be used as a contrast agent in an MRI or heating source in localised hyperthermia therapy [11]. Also, Barick and Hassan [12] showed a single-step approach for the synthesis of Gly-passivated IONPs, which chemisorbed onto the surface of magnetite NPs through the carboxylate group, leaving amine groups free on the surface. These NPs showed good colloidal stability and biocompatibility with human cancer cell lines (HeLa) and can be used for the hyperthermia treatment of cancer. Another attractive AA is lysine (Lys), which was used as the second stabilising layer (see Zhang et al. [13,14,15]). The prepared NPs were absorbed into the cytoplasm and exert relatively high cytotoxicity. The electrokinetic characterisation of magnetite NPs functionalised with Lys, Gly, and other AAs was discussed by Viota et al. [16], while Park et al. presented their study of the surface charge effect of AAs on the colloidal stability of magnetite NPs [17].

Marinescu et al. described a one-step synthesis of AA-functionalised magnetite NPs using the reaction of Fe(III) and Fe(II) precipitation with ammonia solution in the presence of the AAs, in particular tryptophan (Trp) [18]. It was shown that at pH > 10, Trp attaches to the magnetite nanoparticle surface involving bidentate chelation of carboxylate AA groups. Another study focused on the characterisation of the adsorption processes of Trp on the surface of Fe_3_O_4_ modified by ionic liquid [C8MIM][Br] [19]. The adsorption of L-Trp and other AAs on the surface of magnetic silica NPs, which were modified by grafting with carboxymethyl-*β*-cyclodextrin (CMSD) via carbodiimide activation, has also been reported [20]. According to Ghosh et al., Fe_3_O_4_-SiO_2_-CMCD-MNPs can selectively adsorb AA enantiomers through host–guest interaction and could have potential applications in chiral separation technology [20]. Antosova et al. found that Gly-, Lys- and Trp-coated MNPs have a myriad of effects on amyloid fibrils and can destroy lysozyme, α-lactalbumin, and insulin amyloid fibrils in a concentration-dependent manner [21,22].

In the present work, a three-step approach for the preparation of functionalised MNPs is presented. In the first step, MNPs were prepared by the co-precipitation method, while the second step is related to the stabilisation of the NPs with perchloric acid HClO_4_ (MNPs), and the third step consists of the MNP functionalisation by three types of AAs: positively charged polar Lys, non-polar aliphatic Gly, and aromatic Trp. The chemical structure and some selected properties of the AAs used are shown in Table 1.

Moreover, the prepared samples were characterised in terms of their morphology, size distribution, and magnetic properties. Since AA imaging by MRI can provide additional information about pathological metabolism, which can increase the diagnostic potential of MRI methodology, we have focused on the MRI analysis of Gly-, Lys-, Trp-MNPs to investigate changes in the relaxation properties of the MNPs for the three AA attached to the surface of the stabilised MNPs.

## 2. Materials and Methods 

### 2.1. Materials

DL-Lys monohydrochloride (98%), Gly (99%), DL-Trp (99%), ferric chloride hexahydrate (FeCl_3_·6H_2_O), ferrous sulphate heptahydrate (FeSO_4_·7H_2_O), ammonium hydroxide (NH_4_OH), and perchloric acid (HClO_4_) were purchased from Sigma-Aldrich (St. Louis, MO, USA).

### 2.2. NP preparation

**Figure 1:**
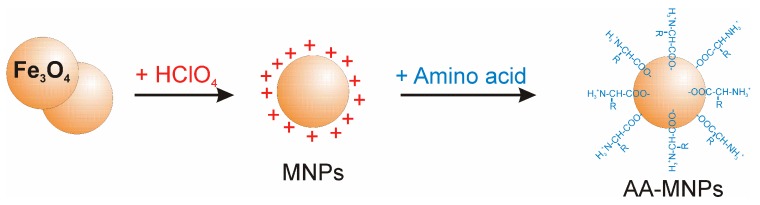
The scheme of magnetic nanoparticle (MNP) functionalisation by amino acids (AAs).

Aqueous solution of magnetic NPs stabilised with perchloric acid was prepared by the Massart’s co-precipitation method [23], with slight modifications as described in [21,24,25]. Iron oxide NPs (IONPs) were prepared by co-precipitation of ferric and ferrous salts (molar ratio 2:1) in the basic solution of 25% NH_4_OH under constant stirring. The suspension was washed four times with water to reach a pH of approximately 9 [26,27,28]. Then, IONPs were stabilized by perchloric acid, which resulted in oxidation of magnetite to maghemite (*γ*-Fe_2_O_3_). Finally, electrostatically stabilised MNPs were centrifuged at 35,000 rpm for 120 min to remove free HClO_4_ molecules. The concentration of IONPs in magnetic fluid, estimated by thiocyanate colorimetry [29], was 30 mg/mL. The coating of MNPs with Gly, Lys, and Trp was carried out by mixing the aqueous solution of the corresponding AA with MNPs in the weight ratios of AA/IONPs = 5, 2, and 8, respectively. The optimal ratios were selected on the basis of our previous optimisation experiments [21,22,24]. After a 72-hour mixing, the samples were centrifuged to remove free AA molecules; the nonmagnetic fraction (supernatant) was separated and withdrawn. The sediment (magnetic fraction) containing AA–MNPs (Figure 1) was used for further experiments.

### 2.3. Characterisation

In this article, we provide comprehensive information on the particle size, size distribution, and NP shape, which requires the use of more than one characterization technique. For this purpose, the dynamic light scattering (DLS) method, transmission electron microscopy (TEM), scanning electron microscopy (SEM), and magnetic measurements (MAG) were used.

The particle size distribution and the average particle size of uncoated MNPs and AA–MNPs were determined at 25 ± 0.1 °C using a Zetasizer Nano ZS apparatus (Malvern Instruments Ltd., Malvern, UK) operating in backscattering mode at an angle of 173°. The dispersions were diluted to get an optimal intensity of ~10^5^ counts per second; thus, the samples contained approximately 100 mg/L of IONPs and their pH varied between 2.88 and 4.6 in dependence of AA used. The aggregation state of the NPs in the aqueous dispersions was characterised by the intensity average hydrodynamic diameter (*D_DLS_*) values and by the polydispersity index (PDI = *D_w_*/*D_n_*). The zeta potential measurements were performed using the same Zetasizer Nano ZS device to determine NP surface charge. Zeta potential serves not only as an indicator of the interaction between magnetic NPs and AA, but also provides us with information regarding the stability of the prepared samples.

TEM has been extensively employed for the characterisation of structures and the analysis of advanced materials. A TEM JEOL JEM 2100F UHR microscope was used to observe the morphology of the studied samples. SEM (JEOL 7000F microscope, Tokyo, Japan) was applied for the characterisation of the sample morphology of both MNPs and AA–MNPs. The samples were prepared by deposition of the sample containing MNPs and AA–MNPs on glass and dried under vacuum prior to sputtering with gold and subsequent imaging.

Measurements of magnetisation were performed employing a SQUID magnetometer (MPMS 5XL, Quantum Design, Inc., San Diego, CA, USA). The magnetisation curves were measured at room temperature (298 K) in the static magnetic field ranging up to 5 T. The investigated samples were sealed in a plastic sample holder and the diamagnetic signal was subtracted from the total magnetisation. The concentration of Fe_3_O_4_ in AA–MNPs was 25 mg/mL.

### 2.4. MRI

The MRI measurements were performed using a 7 T BioSpec Bruker system. We used three different protocols for *T*_1_, *T*_2_, and *T*_2_* parametric mapping:

- *T*_1_ mapping—Rapid Acquisition with Refocused Echoes (RARE) pulse sequence, with repetition time *TR* = 5500, 3000, 1500, 800, 400 and 200 ms, and echo time *TE* = 7 ms.
- *T*_2_ mapping—Multi-Slice Multi-Echo (MSME) pulse sequence, with repetition time *TR* = 2000 ms, starting echo time *TE* = 8 ms, spacing = 8 ms, and 25 images.
- *T*_2_* mapping—Multi Gradient Echo (MGE) pulse sequence, with repetition time *TR* = 800 ms, starting echo time *TE* = 2.09 ms, spacing = 2.23 ms (uncoated MNPs), 5 ms (coated Gly-, Lys-, Trp-MNPs), and 10 images.

The analysed samples were divided into four groups:

- MNPs: Magnetic fluid with acidic pH containing IONPs stabilised by HClO_4_, without AA coating.
- Lys-MNPs: Magnetic NPs coated with Lys.
- Gly-MNPs: Magnetic NPs coated with Gly.
- Trp-MNPs: Magnetic NPs coated with Trp.

To perform the relaxivity evaluation of uncoated as well as AA-coated MNPs, we prepared the concentration gradient (2.5 × 10^−3^–0.02 mg/mL) of the IONPs core. First, the signal intensity values were acquired and evaluated as the relative contrast (RC). The RC of IONPs as a negative contrast agent (*I*_0_ > *I*) is defined as follows:

RC = (*I* − *I*_0_)/*I*_0_, (1)

where *I_0_* is the signal intensity without IONPs, and *I* represents the signal intensity with IONPs.

Subsequently, we determined the longitudinal and transversal relaxation times (*T*_1_, *T*_2_, and *T*_2_*) of the samples, by fitting with the following functions:

*M(t*) = *A*+*M*_0_*(1 − exp(*t*/*T*_1_)), (2)

*y* = *A*+C*exp(*−t*/ *T*_2_), (3)

where *M*_0_ is the equilibrium magnetisation, *A* is the absolute bias, *T*_1_ is the longitudinal recovery time, *C* is the signal intensity, and *T*_2_ is the transversal relaxation time. The value of *T*_2_ is influenced only by atomic molecular interactions, while the T_2_* value reflects atomic molecular interactions as well as the main magnetic field (*B*_0_) inhomogeneities. Finally, we calculated and evaluated the transversal and longitudinal relaxation rates (*R*_1_, *R*_2_, and *R*_2_*) and relaxivity (*r*_1_, *r*_2_, *r*_2_*) values. The transversal relaxation rate (*R_n_*) is inversely related to the relaxation time (*T_n_*):

*R_n_* = 1/*T_n_* (*n* = 1 or 2) (4)

The change in *R_n_*, which characterises the efficiency of magnetic particles for contrast properties in MRI, is defined as the relaxivity of the particle (contrast agent):

*r_n_* = (*R_n_* − *R_n_*^0^)/*C_MP_* (*n* = 1 or 2) (5)

where *R_n_*^0^ is the relaxation rate in the absence of magnetic particles, *R_n_* represents the relaxation rate in the presence of magnetic particles, and *C_MP_* is the magnetic particle concentration. 

We employed the Paravision 6.0.1 Image Sequence Analysis tool (Bruker, Billerica, MA, USA) and Matlab R2011b software tool (Mathworks Inc., Natic, MA, USA) for data processing.

## 3. Results and Discussion

### 3.1. Physical Characterisation

In our previous papers [21,22,24], we identified the optimised conditions for the adsorption of AAs on MNPs and characterised their physico-chemical properties. In these studies, the prepared samples were characterised in terms of the weight ratio of the AA covering and the mass of IONPs by thermogravimetric and differential thermal analyses. Fourier transform infrared and X-ray photoelectron spectroscopy provided information on the chemical bonds between the IONPs core and the organic surface coverage. 

In this study, we have added the results from TEM observation as well as from DLS, and we have completed the results from magnetic measurements for the sample MNPs, Gly-MNPs, Lys-MNPs and Trp-MNPs with the following optimal weight ratios AA/IONPs = 5, 2, and 8, respectively.

**Figure 2:**
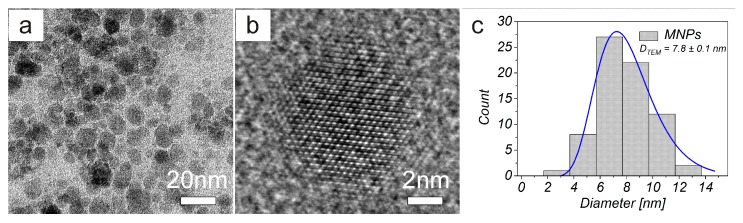
(**a**) TEM and (**b**) high resolution transmission electron microscope (HRTEM) images of MNPs. (**c**) TEM size distribution obtained from TEM images of MNPs.

**Figure 3:**
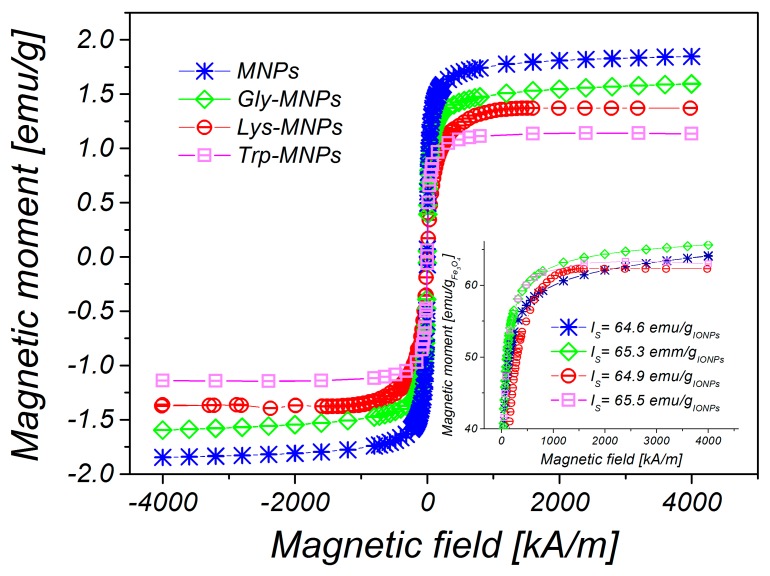
Magnetisation curves of uncoated MNPs, Gly-, Lys- and Trp-magnetic nanoparticles (MNPs) with concentration *c* = 25 mg iron oxide nanoparticles (IONPs)/mL. Inset: recalculated saturation magnetization of the samples on emu/g_IONPs_.

Figure 2 shows the TEM image of MNPs (a) and high resolution transmission electron microscope (HRTEM) image analysing crystal structure of magnetic nanoparticle on an atomic level (b). The prepared MNPs were roughly spherical in shape with a size ranging from 5 nm to 16 nm. The magnetic core mean diameter (*D*_TEM_) was equal to 7.8±0.1 nm (c). We also measured the hysteresis loops of all prepared samples, where each of the curves exhibits zero hysteresis. Therefore, both MNPs and AA–MNPs were considered to be in the superparamagnetic regime. The saturation magnetisations of samples were found to be 1.76, 1.50, 1.37, and 1.15 emu/g for the uncoated, Gly-, Lys- and Trp- samples, respectively (Figure 3). After recalculating the values per IONPs amount, the saturation magnetization of MNPs, Gly-MNPs, Lys-MNPs, and Trp-MNPs were 62.4, 63.1, 62.7, and 63.3 emu/g_IONPs_, respectively (Figure 3 inset). The values were very close and were in the range of values (30 to 80 emu/g) reported in the literature (at 300 K) for synthetic IONPs fine powders [30].

**Figure 4:**
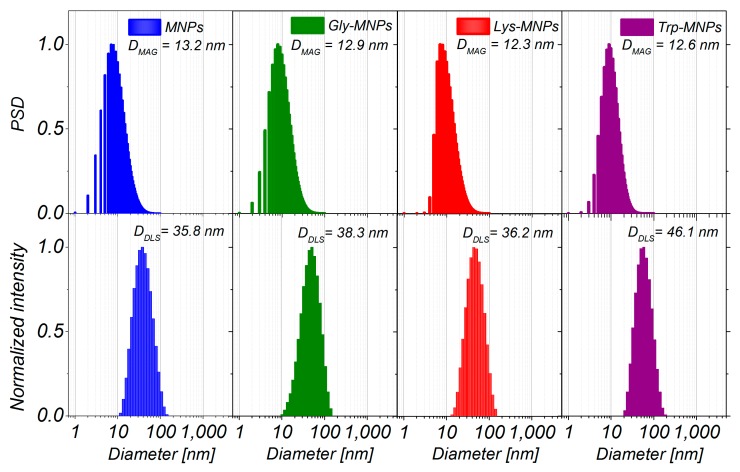
The particle size distribution of magnetic cores obtained from magnetic measurements (upper row), and the size distribution of MNPs, Gly-MNPs, Lys-MNPs, and Trp-MNPs obtained by dynamic light scattering (DLS) technique (lower row).

As can be seen from Figure 3, the magnetisation decreases with increased coating layer thickness. This result can be explained by the fact that magnetisation is proportional to the amount of magnetic material. Increasing the coating layer thickness increases the nonmagnetic material amount (AA) of the sample. Therefore, the greater the amount of the coating material, the lesser the amount of IONPs in the same weight of the sample. After fitting of magnetisation curves by the Langevin function, the magnetic core diameters (*D*_MAG_) were calculated (Table 2). The obtained core diameters (*D*_MAG_) are almost identical, indicating that the coating layer does not influence the core diameter of MNPs. Corresponding size distributions of magnetic core diameters are presented in Figure 4. 

**Figure 5:**
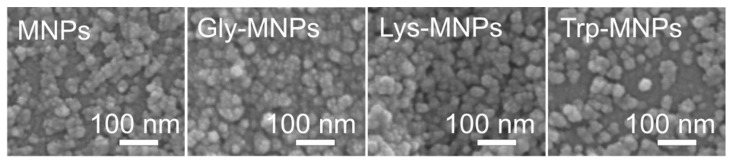
Scanning electronic microscope (SEM) images of MNPs, Gly-MNPs, Lys-MNPs, and Trp-MNPs.

The morphology of uncoated MNPs, Gly-MNPs, Lys-MNPs, and Trp-MNPs was observed using SEM, and the obtained images are presented in Figure 5. The SEM images suggest that both MNPs and AA–MNPs are roughly spherical with smooth surface and have a high level of polydispersity. Histograms of the particle size distributions are constructed from the data obtained from SEM images (Figure 4) and the mean diameters were obtained from the log-normal fit of histograms (*D*_SEM_ = 26.4 ± 0.4, 27.4 ± 0.2, 31.1 ± 0.3, and 37.4 ± 0.9 nm for the uncoated, Gly, Lys, and Trp samples, respectively). The increase of NP size (*D*_SEM_) in comparison with uncoated MNPs can be attributed to the AA coating on the MNP surface and also correlates with the increase of the AA´s molecular weight (see Table 2).

**Figure 6:**
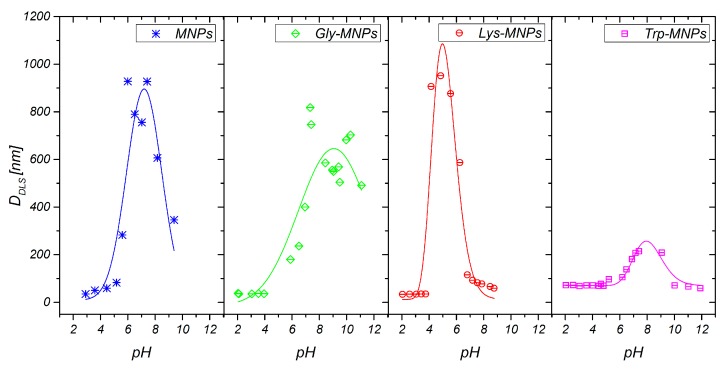
pH-dependent hydrodynamic nanoparticle diameters of MNPs and AA–MNPs.

The DLS method was used to characterise the MNPs and AA–MNP colloid suspensions, from which the samples for microscopy were prepared. This technique is very different from the imaging of dried samples and is sensitive to dynamic aggregation, agglomeration, and so forth. Furthermore, the DLS technique allows the calculation of the hydrodynamic size of MNPs indirectly through the determination of the frequency of movement and modeling of the size from this data. Thus, one can expect different results from this technique compared to the data from microscopic ones. Despite this, the size distributions of the MNPs coated with Gly, Lys, and Trp measured by the DLS were very similar to the SEM results. The mean hydrodynamic diameters (*D*_DLS_) of the prepared samples were 35.8 ± 0.3, 38.3 ± 0.3, 36.2 ± 0.4, and 46.1 ± 0.4 nm for the MNPs, Gly-MNPs, Lys-MNPs, and Trp-MNPs, respectively. The measured hydrodynamic size corresponds to the inorganic NP core with the surface layer that moves in the suspension, whereas the TEM only measures the inorganic core size [31]. Furthermore, the zeta potential measurements (conducted at pH ranging from 3.6 to 4.6, see Table 2) show that in the absence of AA, the zeta potential of MNPs was approximately + 30 mV. This value increased in the presence of AA, suggesting that at least part of the AA was surface-adsorbed (see Table 2). The zeta potential is also a crucial parameter in determining the stability of colloid suspension systems. While the measured values of the AA–MNP samples were above the + 30 mV value in the pH range from 2 to 4, indicating good colloidal stability, Lys-MNPs and Trp-MNPs exhibited good colloidal stability at higher pH values as well (Figure 6).

**Figure 7:**
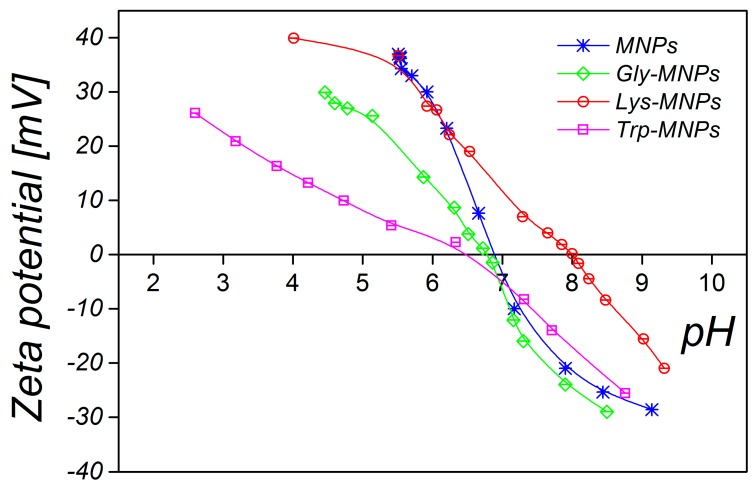
pH-dependent zeta potential of MNPs and AA–MNPs.

In addition, zeta potential measurement as a function of pH has been performed to confirm the surface charge properties and the presence of AAs on the surface of MNPs. Figure 7 shows that the naked MNPs and AA–MNPs were positively charged at lower pH and negatively charged at higher pH. Moreover, the isoelectric point (IEP) of the MNPs increased from pH 6.9 to 8.0 with Lys; in the case of Gly and Trp, IEP slightly shifted towards lower IEP values 6.4 and 6.6. The behaviour is in agreement with the assumption that the more AA molecules adsorbed, the more the IEP moves from the IEP of the MNPs toward the IEP of the pure AAs [32].

### 3.2. MRI analysis

To investigate the relaxivity properties of AA–MNP complexes, we measured and analysed the concentration gradient (2.5, 5, 7.5, 10, 12.5, 15, 17.5, and 20 μg/mL) of each sample: MNPs, Lys-, Gly-, and Trp-MNPs.

The aim was to find the changes in longitudinal, as well as in transversal relaxivity values, caused by different AA binding on the surface of MNPs, which would have definite potential in biomedical applications. The relaxivity is a measure of the sensitivity of the substance (contrast agent) to MRI contrast. Before the relaxivity calculation, the longitudinal and transverse relaxation times were acquired. 

**Figure 8:**
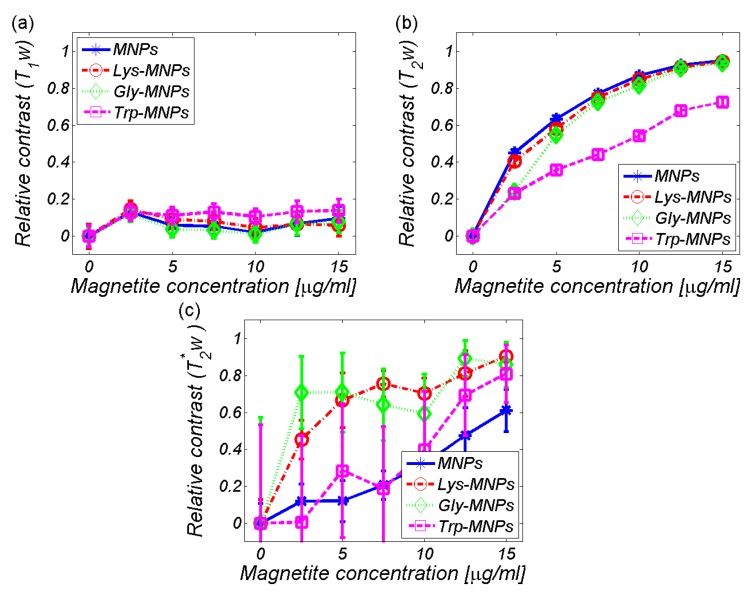
Relative contrast (RC) of the AA–MNP complexes depending on the IONPs/Fe concentration. (**a**) *T*_1_-weighted, (**b**) *T*_2_-weighted, (**c**) *T*_2_*-weighted.

To obtain the relaxation time (*T*_1_, *T*_2_, and *T*_2_*), as well as the signal intensity (*I*_0_ and *I*) values, we used relaxation time-mapping pulse sequences: RARE - *T*_1_, MSME - *T*_2_, and MGE - T_2_*. The primary parameter of substance characterisation in MRI is the RC. The RCs of AA–MNP complexes as well as MNP without AA coating are shown in Figure 8. It is evident that MNPs with and without AA coating significantly affect the transversal relaxation time *T*_2_ (Figure 8b) in comparison with the longitudinal relaxation time *T*_1_ (Figure 8a). The data points to the predominant transverse relaxation mechanism that involves mainly spin–spin interaction without the flow of energy. On the other hand, longitudinal relaxation *T*_1_ represents the transfer of energy between spins and their surroundings. However, in both cases, the Trp-MNP complex was noticeably different from the others, although in the *T*_2_-weighted RC it was in a significantly different scale (Figure 8b) and in reverse order as in the *T*_1_-weighted RC (Figure 8a). A different situation was found in the *T*_2_*-weighted RC (Figure 8c), where the plot showed a small change in the RC of Trp-MNP complex in comparison with the MNP without coating (blue line), but a significantly higher error (magenta line). The increase in standard deviation was seen in all samples, suggesting the weighting influence of the magnetic cores of the MNP complexes to the gradient echo acquisition protocol. Indeed, the gradient echo pulse sequence is sensitive to magnetic field inhomogeneities produced by magnetic moments of MNP compounds. The *T*_2_*-weighted images reveal artefacts produced by pure molecular interactions of magnetic moments that only affect the relaxation time of the surrounding protons (pure *T*_2_ relaxation), as well as distortions caused by the main magnetic field inhomogeneities produced by magnetic particles. By comparing Figure 8b,c, we can visualise the scope of this distortion in selected concentrations. Therefore, the pulse sequences based on gradient echo acquisition can display much lower levels of magnetic compounds.

**Figure 9:**
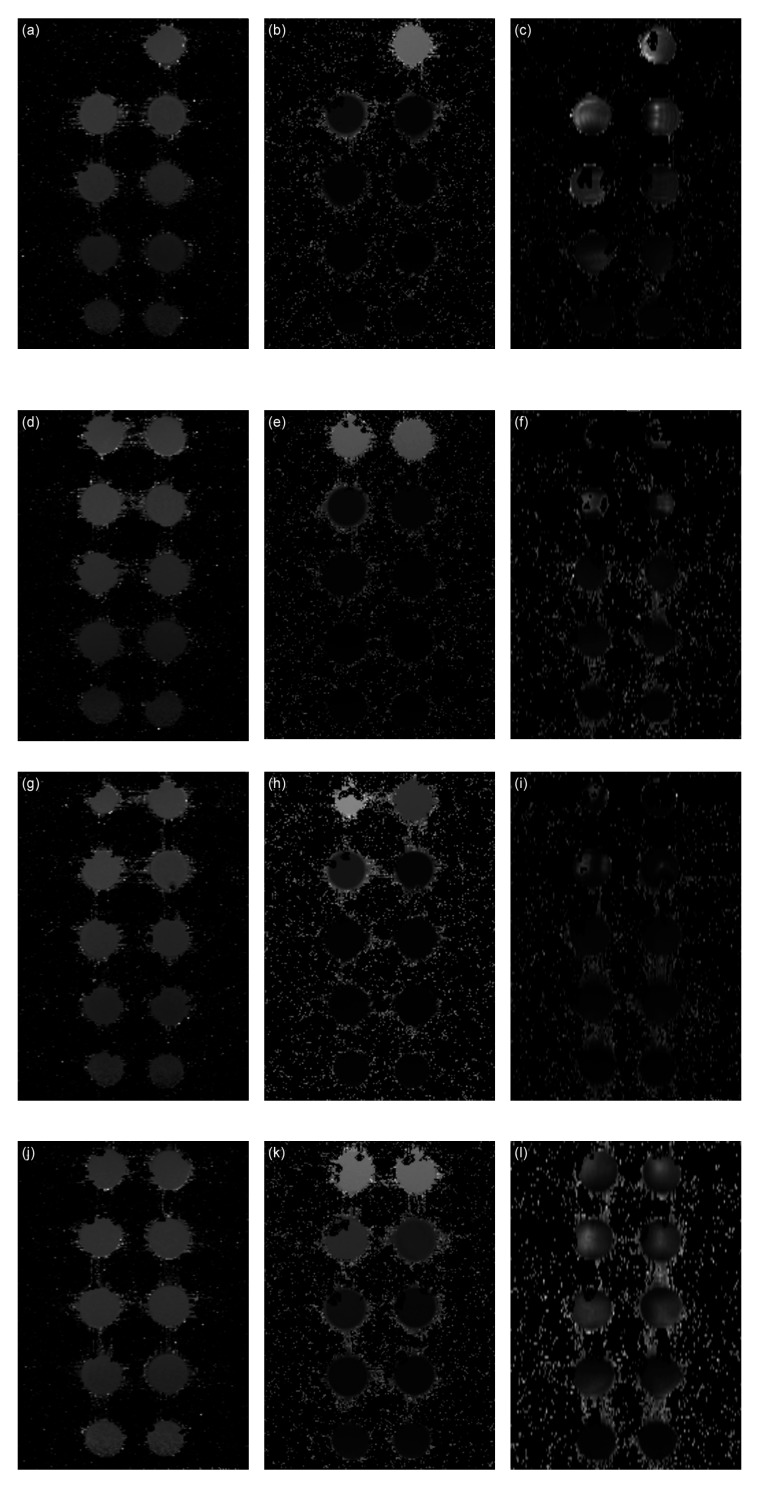
Relaxation time maps of the AA–MNP complexes. (**a**) MNPs *T*_1_ map, (**b**) MNPs *T*_2_ map, (**c**) MNPs *T*_2_* map, (**d**) Lys-MNPs T_1_ map, (e) Lys-MNPs T_2_ map, (**f**) Lys-MNPs T_2_* map, (**g**) Gly-MNPs T_1_ map, (**h**) Gly -MNPs T_2_ map, (**i**) Gly -MNPs T_2_* map, (**j**) Trp-MNPs T_1_ map, (**k**) Trp -MNPs T_2_ map, (**l**) Trp -MNPs *T*_2_* map.

The artefacts caused by field inhomogeneities induced by the IONPs can be clearly seen when comparing the *T*_2_ and *T*_2_* maps in Figure 9. Distortions are visible as "ghost" artefacts in the *T*_2_* maps, which are not apparent in the *T*_1_ and *T*_2_ maps.

**Figure 10:**
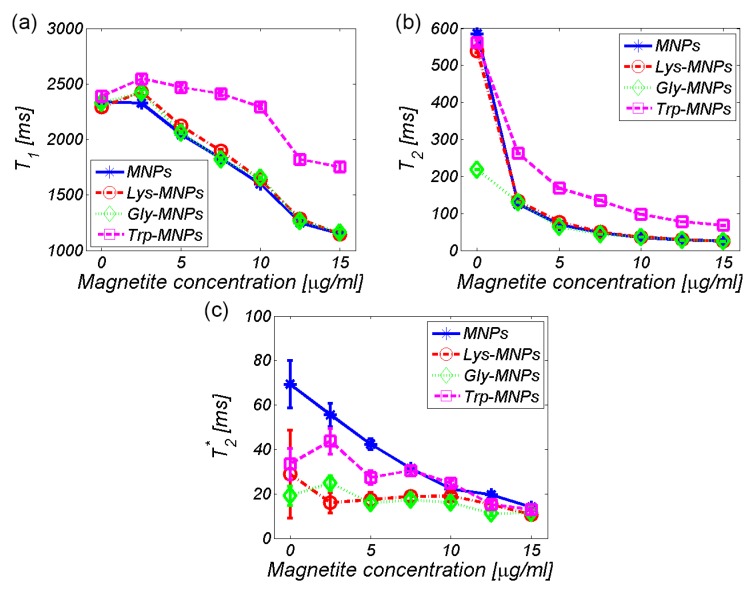
Relaxation time of the AA–MNP complexes depending on the IONP/Fe concentration (**a**) *T*_1_, (**b**) *T*_2_, and (**c**) *T*_2_*.

The relaxation time values from the maps in Figure 9 are quantitatively shown in Figure 10. Theoretically, IONPs should shorten both the longitudinal and transversal relaxation times [33]. Figure 10, in general, confirms this theoretical assumption by decreasing both the relaxation values with an increase in IONPs concentration, although we can see a few exceptions. For the lowest concentration of IONPs (2.5 μg/mL), the increase in longitudinal relaxation time (*T*_1_) was observed for all MNP complexes (Figure 10a). This is a typical feature of positive contrast agents observed under specific conditions, but also in negative contrast agents, including IONPs [33]. The specific conditions involve the size and concentration of IONPs [34]. However, surprisingly, the increase in the relaxation time *T*_2_* value for the Trp- and Gly-MNP complex was also observed, which again was only for the lowest IONPs concentration (Figure 10c). The reason for this remains unclear.

**Figure 11:**
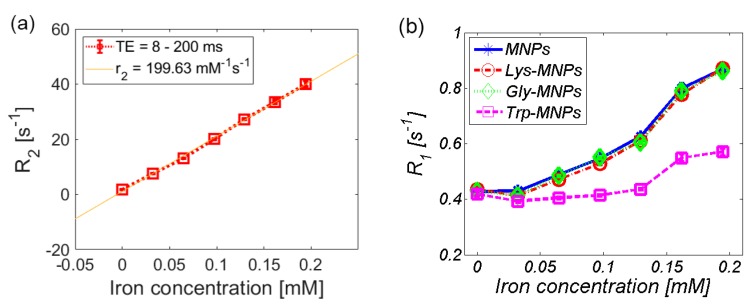

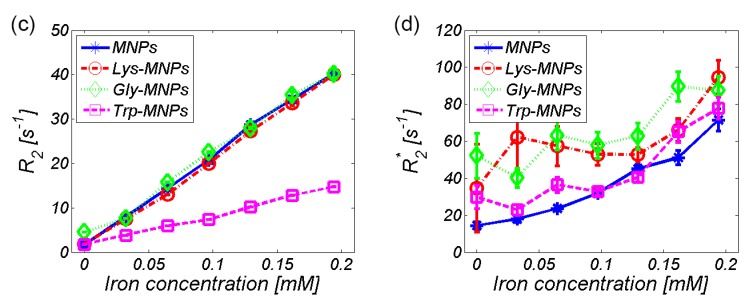
Relaxation rate of the AA–MNP complexes depending on iron concentration. (**a**) Linear fit of the transverse relaxation rate (*R*_2_) that determines the relaxivity value (r_2_) of the Lys-MNP complex. Relaxation rates (**b**) *R*_1_, (**c**) *R*_2_, and (**d**) *R*_2_*.

Both the longitudinal and transverse relaxivity, *r*_1,_ and *r*_2_, were obtained by linear fitting of the longitudinal and transverse relaxation rate, *R*_1_ and *R*_2_, as shown in Figure 11a for the Lys-MNP complex. For relaxivity values determination of all complexes quoted in Table 3, please see Supplementary Material. Figure 11b–d compares the relaxation rates *R_1_*, *R*_2_, and *R*_2_* of different MNP complexes. 

**Figure 12:**
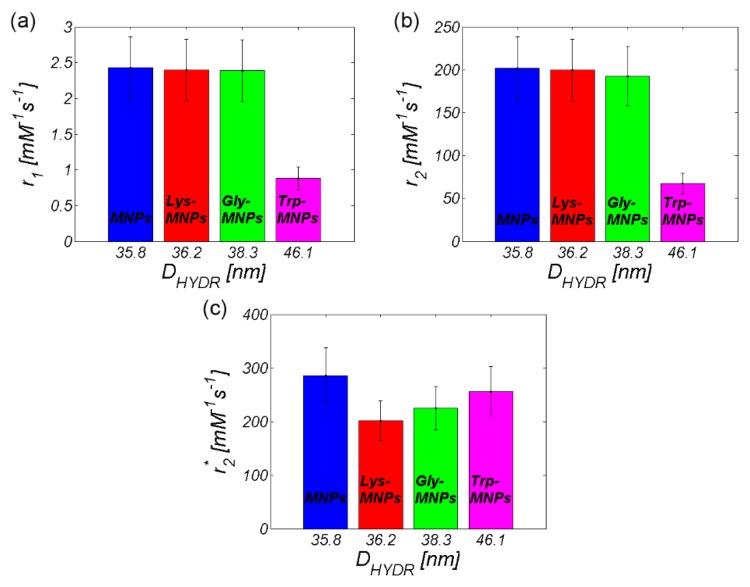
Relaxivity of the AA–MNP complexes depending on hydrodynamic diameter (**a**) *r_1_*, (**b**) *r*_2_, and (**c**) *r*_2_*.

As seen already in the RC comparison (Figure 8), the Trp-MNP complex exhibited significantly different values in comparison with the other complexes, regardless of AA coating. The calculated relaxivity values of the studied AA–MNP complexes are shown in Table 3 and graphically in Figure 12.

Table 3 and Figure 12 also contain information about the hydrodynamic size of AA–MNP complexes from DLS measurement. From the data, it is clear that, in the case of relaxivities, the *r*_1_ and *r*_2_ values decreased with an increase of the hydrodynamic diameter, which depended upon the AA coating. Although in the case of MNPs without the coating and Lys-, and Gly-coated MNPs the difference was not as obvious, the Trp-coated MNPs revealed the pattern of behaviour. On the other hand, we observed the opposite trend in the “behaviour” of the *r*_2_* value. Here, the relaxivity value of the AA–MNP complex grew with an increase of the hydrodynamic diameter, and, moreover, was lower than the relaxivity of the MNP complex without coating. These findings are surprising, and thus require additional theoretical analysis and further experimental verification. Unfortunately, we could not compare our results with the findings of other groups, since we were unable to find similar outcomes in the available literature. The only suitable option is to compare our values with the relaxivity values of commercially available contrast agents based on iron oxide NPs [35]. However, we realise that this comparison is also not ideal as it compares data obtained at different temperatures and magnetic field sizes (Table 4). Moreover, we do not have at our disposal the hydrodynamic size of the particles, although we believe that the first approach is sufficient. 

As shown in Table 4, the values of the longitudinal relaxivity (*r*_1_) were approximately the same in both our AA–MNP complexes and the commercially available contrast agents. The only exception was the Trp-MNP complex with a lower value. A very similar situation was in the case of transverse relaxivity (*r*_2_), but with slightly higher values than commercially available complexes. The Trp-MNP complex was again an exception. Unfortunately, there is no data available to compare the obtained *r*_2_* values against.

**Figure 13:**
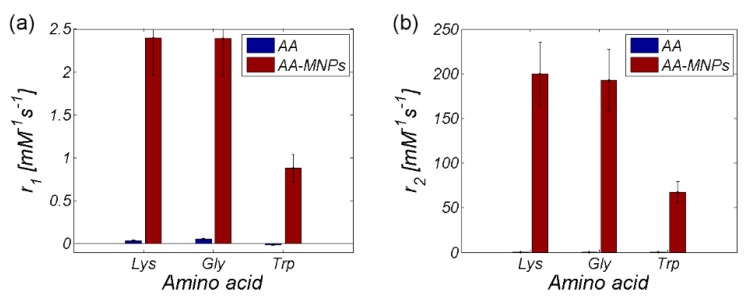
Relaxivity comparison of MNP complexes with and without AA coating (**a**) *r*_1_ and (**b**) *r*_2_.

The data from parametric imaging of pure AA proves the negligible effect of AA alone on the relaxivity of the entire AA–MNP complex (Table 1), which is also illustrated in Figure 13. From this point of view, in our opinion, the only relevant contribution of AA to the relaxivity change of AA–MNP complex is the modification of the hydrodynamic size of the AA–MNP complex (Figure 12). The concentration gradient of AA alone, for relaxivity measurement, was calculated from the concentration gradient of IONP cores with coated AA.

**Figure 14:**
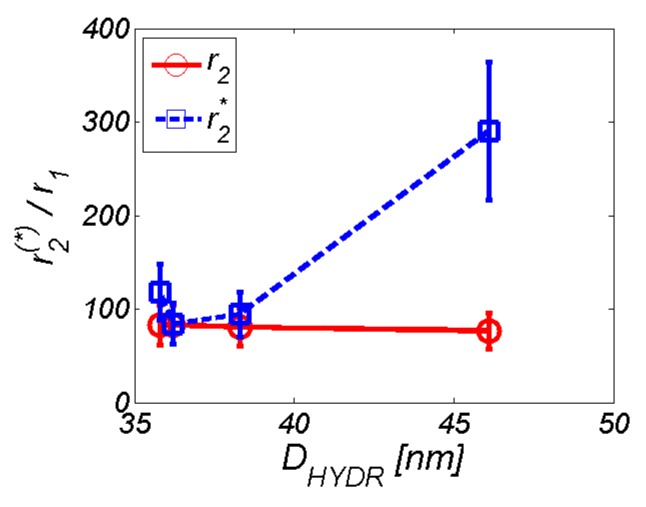
Ratio of transverse and longitudinal relaxivity depending on hydrodynamic diameter.

For practical applications, the ratio of transversal and longitudinal relaxivity was used. It characterises the efficacy of the substance as an MRI contrast agent. High values of *r*_2_^(*)^*/r*_1_ define the dominant *T*_2_ contrast mechanism that is characteristic for hypointensive (dark) artefacts. Conversely, low values of *r*_2_^(*)^/*r*_1_ define the prevailing *T*_1_ contrast mechanism, which is associated with hyperintensive (bright) artefacts. In the case of the studied MNPs coated with AA, it was evident that these complexes behave like a pure *T*_2_ contrast agent (Figure 14). However, we can observe the increasing difference between *r*_2_/*r*_1_ and *r*_2_*/*r*_1_ ratio with increasing hydrodynamic diameter. We suppose that the reason for this difference results from the altered physical background of the *T*_2_ and *T*_2_* weighted sequences. *T*_2_ value is correlated with the representation of the slowly moving fraction of water molecules. The more slowly moving water-binding macromolecules are present in the solution, the lower the *T*_2_ value. On the other side, *T*_2_* value is also related to the non-homogeneities of the static magnetic field, primarily due to the presence of metal ions. Unfortunately, this still does not answer the question of why we do not observe at least a slight increase in relaxivity with increasing hydrodynamic diameter also for the *T*_2_ weighted sequence. It is probably caused by the manner of AA binding to the surface of MNP, as well as by the hydrophilic/hydrophobic balance of organic coating. However, it requires additional molecular dynamics simulations, which are beyond the scope of this paper.

**Figure 15:**
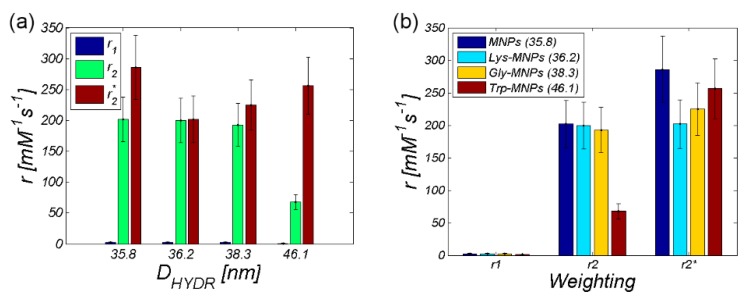
Comparison of the relaxivities of AA–MNP complexes depending on (**a**) hydrodynamic diameter, (**b**) weighting.

For comparative purposes, we also provide the plots with a relaxivity comparison of all AA–MNP complexes, where Figure 15a shows the relaxivity depending on the hydrodynamic diameter, while Figure 15b shows the relaxivity depending on the weighting. Both figures prove the dominant *T*_2_ relaxation mechanism in AA–MNP complexes, as well as pointing to the opposite mechanism of relaxation action at *r*_1_ and *r*_2_ on one side, and *r*_2_* on the other side.

## 4. Conclusions

In this study, we investigated the physico-chemical properties of positively charged magnetic fluids consisting of magnetic NPs functionalised by different AAs: Gly, Lys, and Trp. It was observed that the AAs affect the size of the dispersed particles and the isoelectric point, as well as the zeta potential of MNPs. We showed that the resulting change in hydrodynamic diameter resulted in a modification of the relaxivity value of the AA–MNP complexes. On the one hand, we observed a decrease in relaxivity value (*r*_1_ and *r*_2_) with an increase in hydrodynamic diameter (*r*_1_ and *r*_2_ are comparable with commercially available contrast agents’ relaxivities); on the other hand, we observed an increase in *r*_2_* value with an increase in hydrodynamic size. The reason for this opposite mechanism of relaxation action is not yet apparent, but we assume that it may be caused by the significant sensitivity of *T*_2_* weighted sequences to field inhomogeneities due to the presence of superparamagnetic nanoparticles. However, the presented results provide an attractive preliminary insight into the impact of AA coating on the relaxivity properties of AA–MNP complexes, which has a specific application as a molecular contrast imaging agent based upon the magnetic NPs and magnetic resonance techniques.

## Figures and Tables

**Table 1:** Chemical structure and selected properties of glycine (Gly), lysine (Lys), and tryptophan (Trp).

Name	Structure	Three Letter Code	Molecular Weight	Isoelectric Point (IEP)
Gly (Nonpolar, aliphatic R-group)	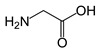	Gly	75	5.97
Lys (Positively charged R-group)	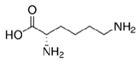	Lys	146	9.74
Trp (Aromatic R-group)	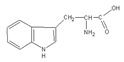	Trp	204	5.89

**Table 2:** Physico-chemical properties of MNPs and AA–MNPs obtained by different techniques. *The pH at which the zeta potential value has been determined.

Samples	*D*_MAG_ (nm)	*D*_TEM_(nm)	*D*_DLS_(nm)	Layer Thickness (nm)	Zeta Potential (mV)	Isoelectric Point pH	*M_s_* (emu/g)
MNPs	13.2 ± 0.2	7.8 ± 0.3	35.8 ± 0.3	−	30.2±1.7 (pH = 3.6)	6.9	1.76
Gly-MNPs	12.9 ± 0.1	8.6 ± 0.1	38.3 ± 0.3	1.25	39.4±1.3 (pH = 3.9)	6.6	1.50
Lys-MNPs	12.3 ± 0.4	8.4 ± 0.1	36.2 ± 0.4	1.40	34.6±2.3 (pH = 4.1)	8.0	1.37
Trp-MNPs	12.6 ± 0.2	8.7 ± 0.1	46.1 ± 0.4	5.15	36.7±1.3 (pH = 4.6)	6.4	1.15

**Table 3:** Relaxivity values of MNP complexes with and without AA coating (Lys, Gly, Trp). Hydrodynamic diameter of complexes obtained by the DLS technique. (Please see Supplementary Material how relaxivities were determined.)

Relaxivity-Diameter	MNPs	Lys	Lys-MNPs	Gly	Gly-MNPs	Trp	Trp-MNPs
*r*_1_ (mM^−1^s^−1^)	2.4 ± 0.4	0.036 ± 0.007	2.4 ± 0.4	0.055 ± 0.01	2.4 ± 0.4	−0.009 ± 0.011	0.9 ± 0.2
*r*_2_ (mM^−1^s^−1^)	201.9 ± 36.3	0.06 ± 0.011	199.6 ± 35.9	0.137 ± 0.025	192.6 ± 34.7	0.025 ± 0.003	67.6 ± 12.2
*r*_2_*(mM^−1^s^−1^)	285.8 ± 51.4	NA	201.8 ± 37.5	NA	225.1 ± 40.5	NA	256.3 ± 46.2
*D*_DLS_ (nm)	35.8 ± 0,3	NA	36.2 ± 0.4	NA	38.3 ± 0.3	NA	46.1 ± 0.4

**Table 4:** Relaxivities’ comparison of our AA–MNP complexes and commercially available contrast agents based on the iron oxide nanoparticles (IONPs).

	*r*_1_ [mM^−1^s^−1^]	*r*_2_ [mM^−1^s^−1^]	Field Strength [T]	Temperature [°C]
Resovist [34]	2.8 ± 0.1	176 ± 9	4.7	37
Feridex [34]	2.3 ± 0.1	105 ± 5	4.7	37
MNPs	2.4 ± 0.4	201.9 ± 36.3	7	22
Lys-MNPs	2.4 ± 0.4	199.6 ± 35.9	7	22
Gly-MNPs	2.4 ± 0.4	192.6 ± 34.7	7	22
Trp-MNPs	0.9 ± 0.2	67.6 ± 12.2	7	22

## Supplementary Materials

The following are available online at https://www.mdpi.com/2079-4991/10/2/394/s1, Figure S1a: Relaxivity *r*_1_ determination of the uncoated MNPs. Figure S1b: Relaxivity *r*_2_ determination of the uncoated MNPs. Figure S1c: Relaxivity *r*_2_* determination of the uncoated MNPs. Figure S2a: Relaxivity *r*_1_ determination of the Lys-MNPs complex. Figure S2b: Relaxivity *r*_2_ determination of the Lys-MNPs complex. Figure S2c: Relaxivity *r*_2_* determination of the Lys-MNPs complex. Figure S3a: Relaxivity *r*_1_ determination of the Gly-MNPs complex. Figure S3b: Relaxivity *r*_2_ determination of the Gly-MNPs complex. Figure S3c: Relaxivity *r*_2_* determination of the Gly-MNPs complex. Figure S4a: Relaxivity *r*_1_ determination of the Trp-MNPs complex. Figure S4b: Relaxivity *r*_2_ determination of the Trp-MNPs complex. Figure S4c: Relaxivity *r*_2_* determination of the Trp-MNPs complex. Figure S5a: Relaxivity *r*_1_ determination of the Lys without MNPs. Figure S5b: Relaxivity *r*_2_ determination of the Lys without MNPs. Figure S6a: Relaxivity *r*_1_ determination of the Gly without MNPs. Figure S6b: Relaxivity *r*_2_ determination of the Gly without MNPs. Figure S7a: Relaxivity *r*_1_ determination of the Trp without MNPs. Figure S7b: Relaxivity *r*_2_ determination of the Trp without MNPs.
