# Supplementary material for: MRI Relaxivity Changes of the Magnetic Nanoparticles Induced by Different Amino Acid Coatings
Source: Nanomaterials (Basel). 2020 Feb 24;10(2):394. doi: 10.3390/nano10020394 (PMC7075310; doi:10.3390/nano10020394)
Supplement: Supplementary file 1 [file nanomaterials-10-00394-s001.pdf]

Article

MRI Relaxivity Changes of the Magnetic Nanoparticles Induced by Different Amino Acid Coatings

Iryna Antal [1], Oliver Strbak [2,*], Iryna Khmara [1], Martina Koneracka [1], Martina Kubovcikova [1], Vlasta Zavisova [1], Martina Kmetova [3], Eva Baranovicova [2] and Dusan Dobrota [3]

[1] Institute of Experimental Physics, Slovak Academy of Sciences, Watsonova 47, 040 01 Kosice, Slovakia; iryna.antal@saske.sk (I.A.); irynakhmara@gmail.com (I.K.); konerack@saske.sk (Martina Koneracka); kubovcikova@saske.sk (Martina Kubovcikova); zavisova@saske.sk (V.Z.)

[2] Biomedical Center Martin, Jessenius Faculty of Medicine in Martin, Comenius University in Bratislava, Mala Hora 4, 036 01 Martin, Slovakia; eva.baranovicova@uniba.sk (E.B.)

[3] Department of Medical Biochemistry, Jessenius Faculty of Medicine in Martin, Comenius University in Bratislava, Mala Hora 4, 036 01 Martin, Slovakia; martinamihalikova09@gmail.com (Martina Kmetova); dusan.dobrota@uniba.sk (D.D.)

* Correspondence: oliver.strbak@centrum.cz; Tel.: +421-43-2633448

Received: 30 December 2019; Accepted: 18 February 2020; Published: 24 February 2020

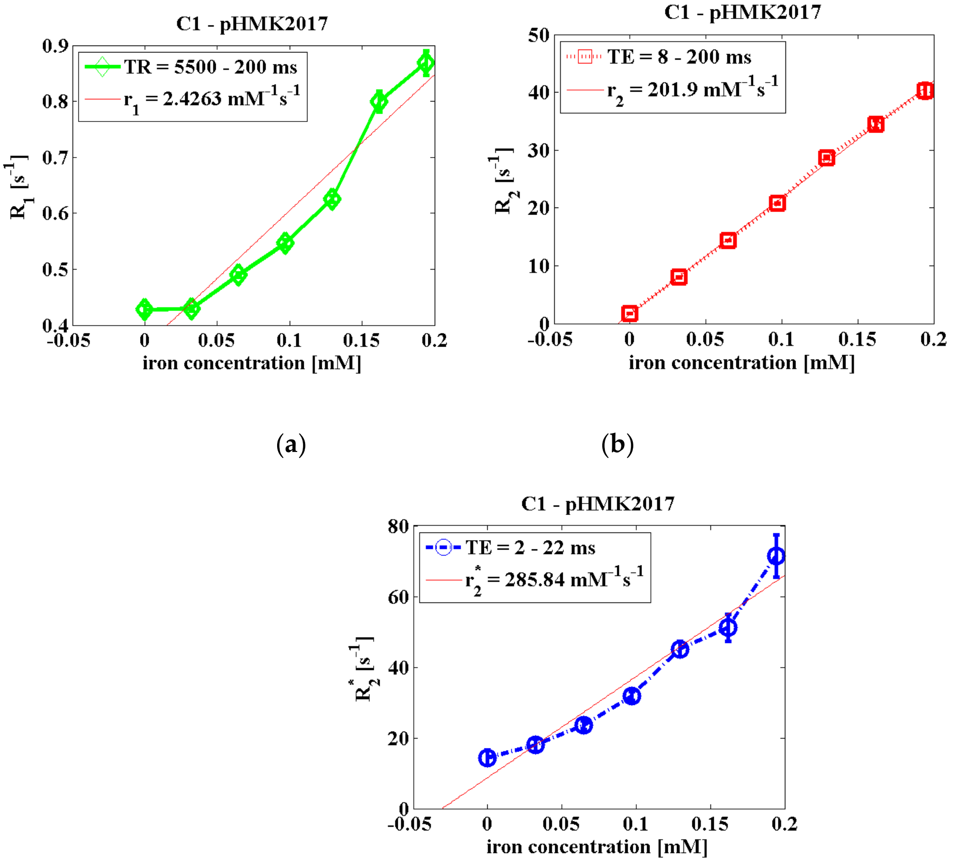

(**a**) (**b**)

(**c**)

Figure S1. (**a**) Relaxivity r_1 determination of the uncoated MNPs. Figure. (**b**) Relaxivity r_2 determination of the uncoated MNPs. (**c**) Relaxivity r_2^* determination of the uncoated MNPs.

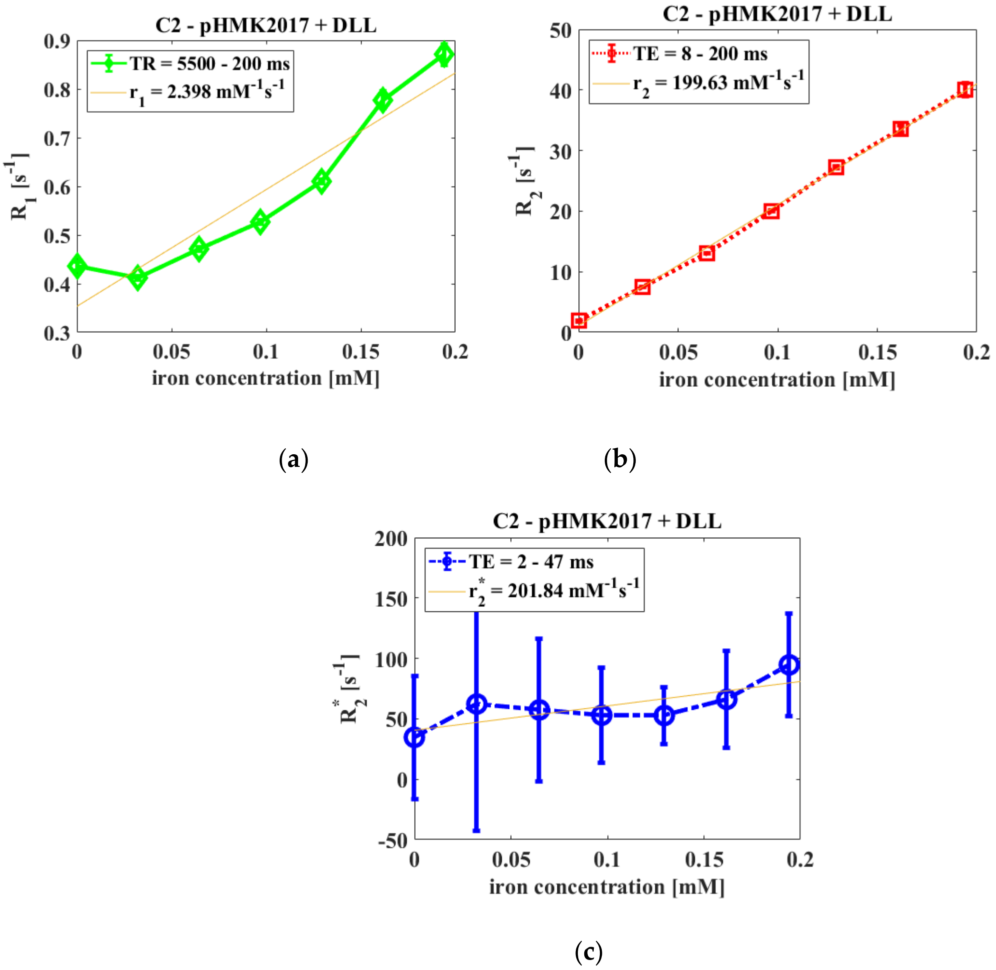

Figure S2. (**a**) Relaxivity r_1 determination of the Lys-MNPs complex. (**b**): Relaxivity r_2 determination of the Lys-MNPs complex. (**c**): Relaxivity r_2^* determination of the Lys-MNPs complex.

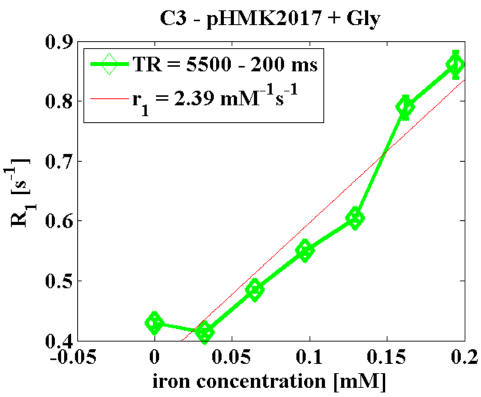

(**a**)

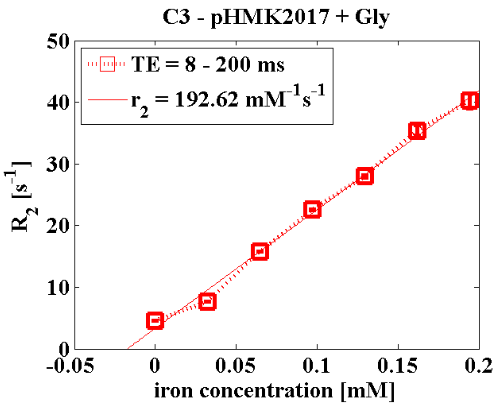

(**b**)

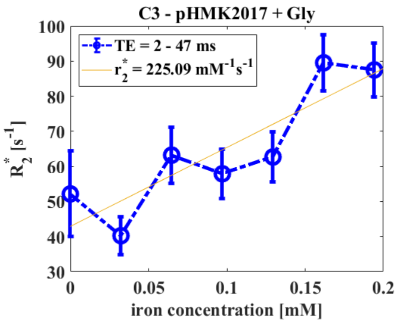

(**c**)

Figure S3. (**a**) Relaxivity r_1 determination of the Gly-MNPs complex. (**b**): Relaxivity r_2 determination of the Gly-MNPs complex. (**c**) Relaxivity r_2^* determination of the Gly-MNPs complex.

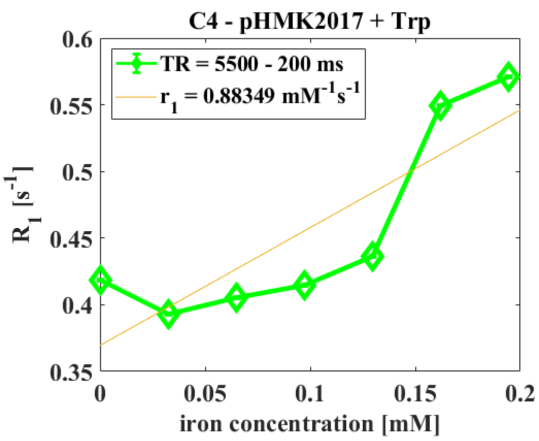

(**a**)

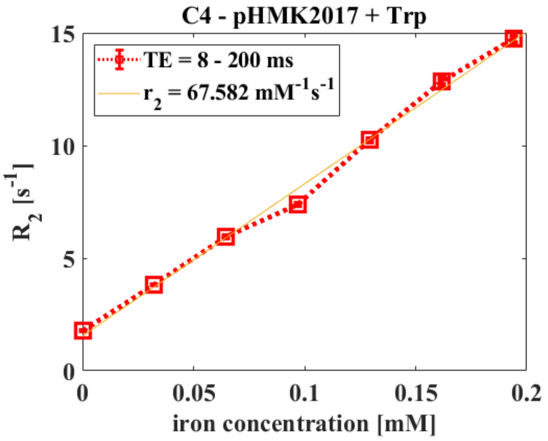

(**b**)

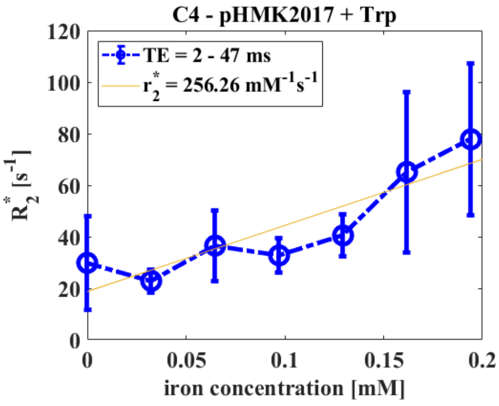

(**c**)

Figure S4. (**a**) Relaxivity r_1 determination of the Trp-MNPs complex (**b**) Relaxivity r_2 determination of the Trp-MNPs complex. (**c**) Relaxivity r_2^* determination of the Trp-MNPs complex.

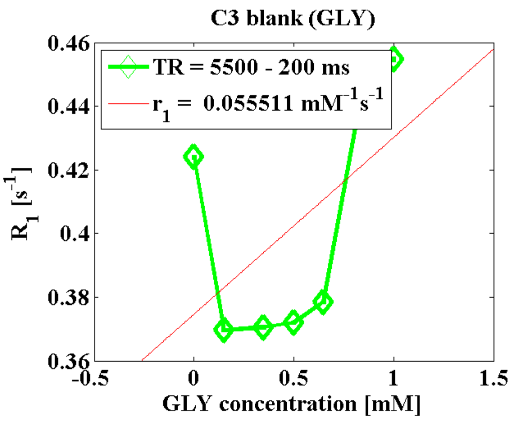

(**a**)

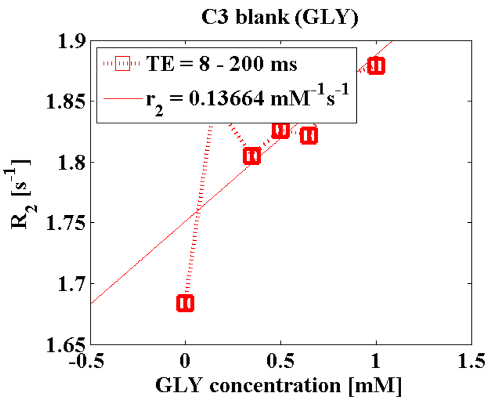

(**b**)

Figure S5. (**a**) Relaxivity r_1 determination of the Lys without MNPs. (**b**): Relaxivity r_2 determination of the Lys without MNPs.

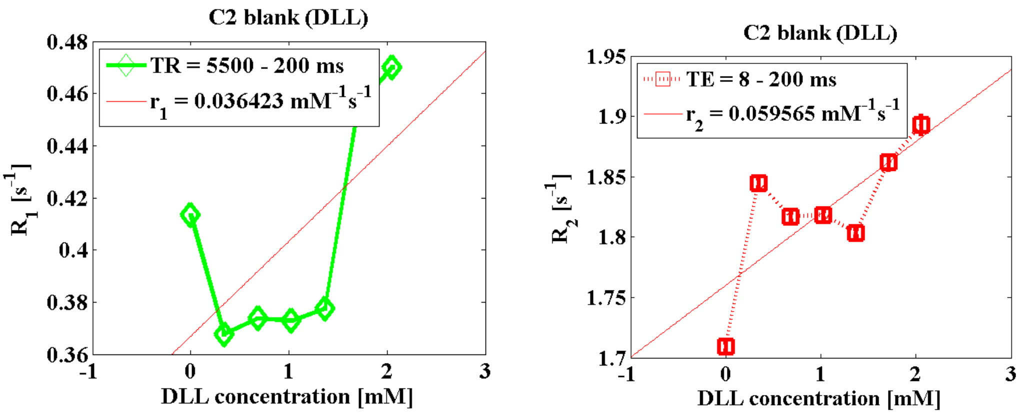

(**a**) (**b**)

Figure S6. (**a**) Relaxivity r_1 determination of the Gly without MNPs. (**b**): Relaxivity r_2 determination of the Gly without MNPs.

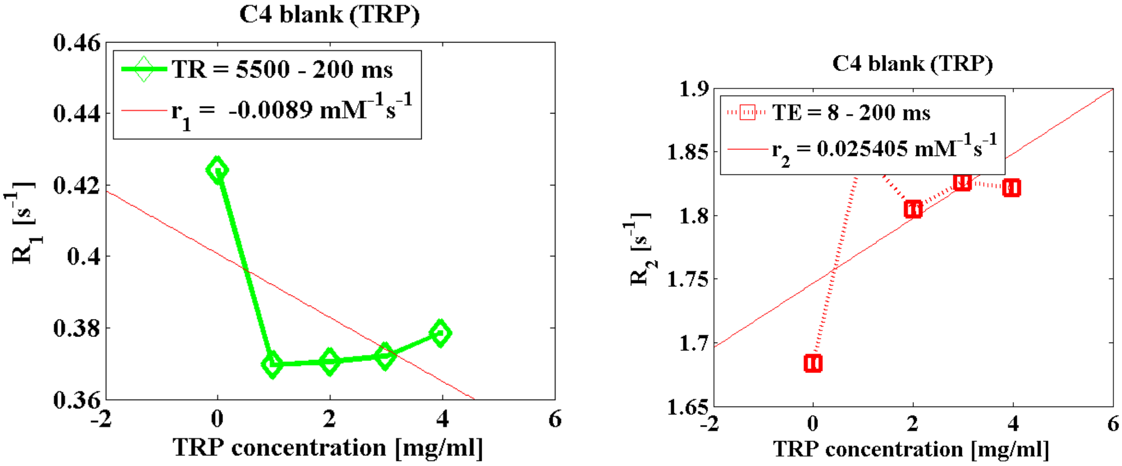

(**a**) (**b**)

Figure S7. (**a**) Relaxivity r_1 determination of the Trp without MNPs. (**b)** Relaxivity r_2 determination of the Trp without MNPs.
